# Brown adipose tissue biology and therapeutic potential

**DOI:** 10.3389/fendo.2013.00014

**Published:** 2013-03-19

**Authors:** Patrick Seale

**Affiliations:** Cell and Developmental Biology, Perelman School of Medicine, University of PennsylvaniaPA, USA

## Brown adipose tissue

**Figure 1:**
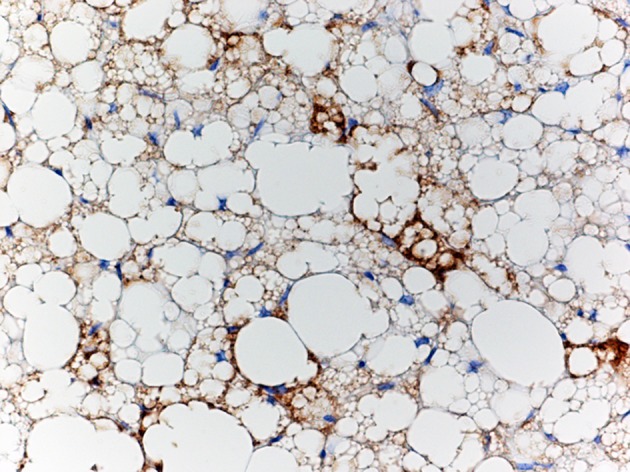
**Brown adipose tissue.** Immunohistochemical staining of Ucp1 expression in brown adipose tissue showing a mixture of large and small adipocytes.

Brown adipose tissue (BAT) (Figure 1) has been recognized as a key thermogenic tissue in rodents for several decades. However, in the last few years, there has been a resurgent interest in the biology and therapeutic potential of BAT. This has been largely driven by a new understanding that most, if not all, healthy adult humans have significant deposits of BAT which can be activated by cold. Moreover, recent insights into the developmental origins of brown adipose cells and the identification of molecules that regulate brown adipocyte activity have provided a conceptual framework for the design of brown fat-based therapies.

Brown fat cells, when activated, are able to take up and oxidize large amounts of fat and carbohydrate for the purpose of producing heat—a process called adaptive thermogenesis. This transformation of chemical energy into heat at the expense of ATP production is mediated by the presence of Uncoupling Protein-1 (Ucp1) in the inner mitochondrial membrane. Ucp1 catalyzes the leak of protons from the intermembrane space back into the matrix, thus reducing the gradient (and its potential energy) used for ATP production.

BAT presumably evolved to protect animals against hypothermia in response to cold exposure. However, BAT is also known to powerfully counteract obesity and metabolic disease, at least in rodents. Numerous studies over recent years revealed that mice with increased amounts of active brown fat are lean, healthy, and able to resist the harmful metabolic effects of high fat diets. Intriguingly, imaging studies in humans show that brown fat activity is reduced in obesity and aging. The field must now address whether the variation in brown fat activity is a cause or consequence of weight gain. Brown fat cells are localized in discrete depots of BAT and are also found clustered amongst white fat cells in white depots. The prevalence and function of these so-called “beige” or “brite” (brown-in-white) cells in humans remains unknown.

There is a growing consensus in the field that brown fat-targeted therapies hold tremendous promise for the treatment of obesity and associated health consequences. It is also increasingly clear that brown fat can function as an effective sink for disposing of excess glucose and fatty acids. This suggests that brown fat-based therapies could be very effective for treating insulin resistance, type-2 diabetes, and dyslipidemia without necessarily reducing body weight.

In this Research Topic, we were able to assemble articles from many of the prominent scientists in the field which focused on many different and important aspects of brown fat biology. The topic begins with a historical perspective (Ricquier, 2012) and also includes reviews and original reports on: test systems to study Ucp1 (Hirschberg et al., 2012); the development of brown adipose cells (Boss and Farmer, 2012; Festuccia et al., 2012; Kozak, 2012; Pisani et al., 2012; Scime, 2012; Yadav and Rane, 2012); the influence of genetics (Kozak, 2012); adrenergic and central control of brown adipocyte activity (Collins, 2012; Morrison et al., 2012); human brown adipose cells and imaging methods (Betz and Enerback, 2012; Hu and Gilsanz, 2012; Muzik et al., 2012; Richard et al., 2012; van Marken Lichtenbelt, 2012); and perspectives for brown adipose-based therapeutics (Betz and Enerback, 2012; Boss and Farmer, 2012). I would like to thank all the contributors and reviewers for their help in putting this interesting and timely collection of articles together.
